# Trial for Alleged Rape

**Published:** 1851-09

**Authors:** 


*Trial for alleged Rape—Curious question of fact—Judge, Jury, and Counsel in a dilemma.* By H. G., M. D., of South Carolina.—At our April term in this district, a man was tried for the crime of rape. The rape was alleged to have been committed in the erect position. The defence was based on the supposition, that it was impossible that such an act could be consummated in that posture; and the jury acquitted the prisoner.

I am not aware that it has ever been attempted to be demonstrated, that a rape could be committed on a woman in the erect position. I infer this, from the fact that no reference was had by counsel, on the trial, to any case of the kind. Chitty, in his Medical Jurisprudence, is silent on this question; nor have I met with anything on the subject, in any work I have read.

The State's attorney admitted he could not conceive how such a thing could be done, but contended that a rape had been clearly proved.

Judge E———, in his charge to the jury, stated, that the finding of guilty, or not guilty, turned entirely on the question at issue. The jury, after an absence of about twenty minutes or half an hour, returned with a verdict of "not guilty."

The woman left the house of the prisoner, where she had been staying all night. She had not gone far before he overtook her; they walked side by side, he embracing her all the time and treating her with great rudeness. Upon crossing a branch, he said he would "do it or die." She replied, she would "die" before he should. Upon this, he confined her left hand under his right arm, with his right arm around her body, and forced her with her back against a tree, in which position the act was consummated as alleged by the woman.

It was proved that the plaintiff had on her back or loins an injury of some kind, which disabled her about three weeks; the plaintiff swore that the hurt was received in the act. This injury could not be accounted for, and was supposed to be a common boil or rising.

It was proposed by counsel to submit the question to physicians, as a question of science, as to whether a rape could be committed in the erect position. The judge objected, saying it was not a question of science, and that any other person could explain as well as physicians.

Now let us inquire, what are the essential conditions necessary to commit a rape in any position? The first and most essential condition is, that the body of the victim be fixed—immovably fixed—so that lateral motion or rotation be impossible, and her hands be so confined that she could not use them to any advantage; these conditions would be indispensable in the horizontal position. Could they be possibly secured in the erect position? Could the act of copulation be consummated in the erect posture by a man and woman, if both were willing? I think no one will deny this. If, then, the woman can be brought by force and against her will, into the necessary posture, and the other essential conditions secured, there is no reason to deny the possibility of the consummation of such an act.

In the horizontal position the ground would furnish the *point d'appui*, and in the erect position a tree would answer the same purpose. Suppose a man in an attempt to commit a rape in the horizontal position, should find himself foiled in consequence of his inability to fix his victim, or secure the conditions above specified, would he not be greatly aided in his efforts by fixing his hold on any immovable object that might present itself, as a root or bush, &c.? Would not such hold give him much advantage when there was nearly a preponderance of strength on the part of the female? Who has not witnessed wrestlers resort to this means to maintain the advantage they had gained over their antagonist, when without such aid they could not have succeeded in their object.

It was contended to be impossible, that, in the erect position he could have introduced the virile organ without the use of his hands into the vagina. The woman swore, that she felt his "private part in her body." The counsel for the prisoner related an anecdote of "England's Virgin Queen," who, in conversation with one of her courtiers on this indelicate subject, expressed the opinion, that the consummation of such an act was impossible under any circumstances. Taking down a sword and unsheathing it, she requested him to introduce it (the sword) into the scabbard while she held it; every attempt to do so, however, was foiled by an adroit movement of the scabbard. This anecdote, though productive of considerable merriment, contained not a particle of argument, as it excluded one of the essential conditions—viz: the fixedness of the vagina.

In regard to the erect position, it is evident the introduction of the penis into the vagina would be more difficult, other things being equal, than in the horizontal posture; their bodies bearing the same relation to each other erect as horizontal; on the ground they would be parallel, erect they would be the same.

Again, it was contended that it was impossible, that he could hold her in that position till he could accomplish his object. The prisoner was a strong and active man, and could entirely overpower a weak woman, (for she was not a very strong woman,) and keep her still in that position as well as in the horizontal, with the aid which the tree would afford him.

In answer to many questions put to her, in reference to the position of her arms and his, how he held her, &c., she replied that she was so frightened and agitated that she could not recollect all the minutiæ.

It was contended she might have used her right hand in preventing the final consummation of the act; but it must be recollected her right hand, as well as her left, was confined and pressed between him and the tree, which greatly aided him, at the sametime it rendered her arms powerless and useless. If she attempted to extricate her arms, her elbows would come in contact with the tree, and render all attempts of the kind nugatory. If we suppose he passed his arm, or arms, around the tree, which was proved to be a "spruce pine," the tremendous pressure he would be enabled to exert on her body, would have the effect to fix and render her immovable and powerless; while, on the other hand, the support the tree would afford would enable him to maintain the erect position, and accomplish his diabolical design as effectually as on the ground.

The tree, which was a spruce pine, does not grow very large in this region. The bark of this tree grows very thick, and is divided into deep longitudinal fissures, in which he would be enabled to fix his hold, and would afford him all the advantages necessary to enable him to accomplish his object, and that as effectually as in the horizontal position. Upon this principle, also, we are enabled to account for the injury proved to have existed on her back. The rude pressure against the rough bark of this tree would not only aid him in rendering her immovable, but the muscles of her lumbar region would be contused, and inflammation and suppuration would ensue.

Further, it was contended, that the separation of her thighs, and the introduction of his virile member, could not have been effected without the use of his hands. But I cannot conceive how this would be more difficult in the erect posture, other essential conditions having been secured, than in the horizontal; and an argument, based upon such a supposition, would be equally forcible against the possibility of such an act being committed in the horizontal position.

Remarks.—We think, with the writer of the foregoing, that the jury acquitted the prisoner on frivolous grounds; for, although the case, as far as our investigations have been pushed on this subject, is without a parallel in the books; yet we not only believe it possible, but deem it almost as easy to commit a rape in the erect as in the horizontal position, especially under the circumstances detailed in the above instance.—*Ed. South. Med. Sur. Journal.*

